# Android malware detection using hybrid ANFIS architecture with low computational cost convolutional layers

**DOI:** 10.7717/peerj-cs.1092

**Published:** 2022-09-26

**Authors:** İsmail Atacak, Kazım Kılıç, İbrahim Alper Doğru

**Affiliations:** IoTLab, Department of Computer Engineering, Faculty of Technology, Gazi University, Ankara, Turkey

**Keywords:** Malware detection, Mobile security, Convolutional neural network, Fuzzy logic, Permission

## Abstract

**Background:**

Android is the most widely used operating system all over the world. Due to its open nature, the Android operating system has become the target of malicious coders. Ensuring privacy and security is of great importance to Android users.

**Methods:**

In this study, a hybrid architecture is proposed for the detection of Android malware from the permission information of applications. The proposed architecture combines the feature extraction power of the convolutional neural network (CNN) architecture and the decision making capability of fuzzy logic. Our method extracts features from permission information with a small number of filters and convolutional layers, and also makes the feature size suitable for ANFIS input. In addition, it allows the permission information to affect the classification without being neglected. In the study, malware was obtained from two different sources and two different data sets were created. In the first dataset, Drebin was used for malware applications, and in the second dataset, CICMalDroid 2020 dataset was used for malware applications. For benign applications, the Google Play Store environment was used.

**Results:**

With the proposed method, 92% accuracy in the first data set and 92% *F*-score value in the weighted average was achieved. In the second data set, an accuracy of 94.6% and an *F*-score of 94.6% on the weighted average were achieved. The results obtained in the study show that the proposed method outperforms both classical machine learning algorithms and fuzzy logic-based studies.

## Introduction

Today, mobile devices have become indispensable in our daily life. We used to benefit from devices, which were used to communicate with each other, however, now they are used in all our transactions thanks to the high capacity and speed features provided by technology (Bhat & Dutta, 2021). Applications developed for mobile devices not only make our work easier, but also allow us to save time. According to the 2022 report of We are Social Digital, 67% of the world’s population uses mobile devices and the number of users is increasing day by day. Among this rate, 97% of users use smartphones. According to researchers, seven out of 10 mobile users use the Android operating system (We are Social, 2022).

Android is a Linux-based and open source operating system developed by Google (Doğru & Kiraz, 2018). The free and functional structure of the Android operating system plays an active role in the preferences of users (Kumar et al., 2020; Guerra-Manzanares, Bahsi & Nõmm, 2021). The Android operating system has become the target of malware developers due to its market share and open source code. Mobile malware developers aim to generate revenue through unethical or even illegal means. For this purpose, they can steal sensitive information such as identity information, location, and contact list. Additionally, they may install adware that sends SMS and forces users to view the web page for which a link is sent (Bala et al., 2021). Applications to the Android operating system can be installed from the Google Play Store or third-party environments (Kim et al., 2018). In particular, applications installed from third-party media pose a threat to users’ privacy and security. This creates a security problem for Android operating system users. Therefore, there is a need for fast and reliable computer-aided detection systems that enable applications to be analyzed as good or bad before they are loaded.

Static analysis and dynamic analysis methods are used to identify malware (Feng et al., 2018). Static analysis is an analysis method that is performed without installing the application on the device. Dynamic analysis, on the other hand, is a type of analysis that examines the behavior of the application after it is installed (Liu et al., 2020).

When the application is downloaded to the Android system, the user is asked to accept the permission requests necessary for the application to work. Mobile users often ignore and accept these permission requests without knowing the risks involved. This leaves users vulnerable to malicious attackers. The application’s permission requests are contained in the manifest.xml file. Applications that require excessive permissions tend to be malicious (Arif et al., 2021). In the literature, many studies have been done for permission-based malware detection. Especially machine learning-based systems have produced successful results in detecting permission-based malware (Mat et al., 2021; Şahın, Akleylek & Kiliç, 2022; Arslan, Doğru & Barişçi, 2019; Şahin et al., 2021).

In studies using machine learning techniques, the features of benign and malicious applications are needed in order to be able to detect or classify. Correct extraction of these features directly affects classification success. Detection of malware using deep learning methods, which is a sub-branch of machine learning, is among the popular research topics. Deep learning-based detection systems can detect malware with high accuracy (Arslan, 2021; Xiao et al., 2019). Convolutional neural networks (CNNs), which have been widely used in image analysis in recent years, have also achieved tremendous results on images of malware (Lachtar, Ibdah & Bacha, 2020; Kong et al., 2022; Yadav et al., 2022). However, despite the success of deep learning architectures, these architectures are disadvantageous due to the need for more resources, high memory consumption and the number of parameters. While the number of classical machine learning and deep learning-based studies for android malware detection has been quite high in the studies carried out so far, fuzzy logic-based studies are limited (Arif et al., 2021; Altaher, 2017; Afifi et al., 2016; Altaher & Barukab, 2017; Abdulla & Altaher, 2015). Contrary to deep learning, fuzzy logic approaches need few inputs and have the ability to make successful decisions with few inputs. For this reason, permission-based features are generally used in the studies and the number of inputs is reduced by the feature selection process. This has caused the researchers to ignore many permissions that the application requests.

The purpose of this study is to detect malicious android applications quickly and with high accuracy using permission information. In this direction, a hybrid detection system that combines the feature extraction and dimension reduction power of the convolution layers in the CNN architecture and the decision making capability of fuzzy logic is proposed. The proposed system reduces the number of inputs for classification by applying feature extraction with only two convolution layers, two pooling layers and five connected layer neurons to all permission information obtained through static analysis. In the last stage, it uses the Adaptive Neuro-Fuzzy Inference System (ANFIS) model to classify applications based on features.

The contributions of the study can be summarized as follows:

• Contrary to other fuzzy logic-based studies (Altaher, 2017; Altaher & Barukab, 2017; Abdulla & Altaher, 2015), permission features are not neglected and all permissions are used.

• The feature extraction power of the convolution layers in the CNN architecture is combined with the decision-making capability of the ANFIS architecture.

• The proposed model achieves better results than similar studies based on fuzzy logic.

• The proposed model has high accuracy and few parameters.

• No similar model has been found in the studies carried out so far.

• In the study, the classification results and the estimated values for each membership function of the proposed model are given separately.

• The results obtained in the study were compared with similar studies. At the same time, the dataset used in the study was classified with classical machine learning techniques and the results were given comparatively.

The remainder of the article is structured as follows. In “Literature review”, static and dynamic analysis methods are explained and a summary of past studies based on machine learning is presented. In “Material & Method”, the details of the proposed method, data set, preprocessing, feature extraction process, classification model and evaluation metrics are explained. In “Results and Discussion”, hyperparameters used in the study, classification results and comparisons with different algorithm results are given. At the same time, the study is evaluated and comparisons with similar studies in the literature are presented in tables. In “Conclusion”, the results of the study and recommendations for future studies are given.

## Literature Review

Mobile malware detection has become one of the popular research topics in recent years. In particular, satisfactory results have been obtained in this area with machine learning and deep learning-based studies (Yuan, Lu & Xue, 2016; Feng et al., 2018; Arshad et al., 2018; Tang et al., 2022; Sasidharan & Thomas, 2021; Zhang et al., 2021).There are two types of approaches, static analysis and dynamic analysis, to detect malware (Feng et al., 2018). In both methods, features belonging to the application are obtained, which enable us to determine whether the applications are malicious or benign. In this section, the types of analysis used for mobile malware detection, as well as the techniques that include machine learning and its sub-branches, and malware detection studies are examined.

### Static analysis

Static analysis is an approach based on analyzing the apk file of android applications before installing them on the device. The advantages of the static analysis method are that it is fast and prevents malicious applications from infecting smart devices. On the other hand, this method is limited in dealing with code scrambling techniques and polymorphic malware (Alzaylaee, Yerima & Sezer, 2020). In this analysis method, there are various features such as permissions, Java codes, intentions, network addresses, and texts in the apk file (Feizollah et al., 2015). Researchers are trying to detect android malware by using one of these features (Yang et al., 2021; Cai et al., 2021) or a combination of them (Li et al., 2019; Wang, Zhao & Wang, 2019).

AppPerm Analyzer (Doğru & Önder, 2020) was presented by Doğru and Önder. This tool creates binary and triple permission groups from apps. It then calculates the risk score and the total risk score based on the use of these permissions and permission groups in malicious and good practices.

BERT (Devlin & Chank, 2022) is open source natural language processing software developed and supported by Google researchers. BERT is an acronym for “Bidirectional Encoder Representations from Transformers”. This model can learn the words in the text and the relationships of the sub-words and process the text as a whole. The BERT model can extract text properties of applications and can be used for malware classification (Kale et al., 2022).

### Dynamic analysis

Dynamic analysis is an analysis approach in which features are obtained by examining the behavior of applications when they are run in virtual environments or real devices (Liu et al., 2020). In this approach, researchers generally use system calls (Guerra-Manzanares, Nõmm & Bahsi, 2019; Hou et al., 2016) and network traffic (Lashkari et al., 2017; Arora, Garg & Peddoju, 2014) features. CPU and RAM usage information, running processes, battery statistics, API function calls and other runtime features are also used in the dynamic analysis approach. Dynamic analysis approach is advantageous against polyformic software with encryption techniques. However, the necessity of running the application in a virtual environment or a real device is difficult and takes time (Feizollah et al., 2015).

### Machine learning based studies

Using machine learning techniques, good results have been obtained in malware detection, as in many areas. Some studies in this area are discussed below:

Arslan et al. obtained permission-based features of 6,500 malicious and 900 good applications and classified them with different machine learning algorithms. In the study giving the comparative performance of machine learning algorithms, they achieved 91.95% accuracy with the KNN algorithm (Arslan, Doğru & Barişçi, 2019).

Mat et al. proposed a method for malware detection that classifies permission-based features with Naive Bayes. They extracted permission-based features of 10,000 applications they obtained from AndroZoo and Drebin. The feature selection process was performed by applying the information gain and chi-square methods to the features they obtained. At the end of the study, they achieved 91.1% accuracy with the Naive Bayes method (Mat et al., 2021).

Şahin et al. proposed a linear regression model-based method for detecting malicious applications from permission information. They tested the method they presented on four different datasets and also improved the classification performance by using the ensemble learning method. They obtained 95.6% accuracy with AMD (Wei et al., 2017) data set, 91.87% with Lopez’s (Urcuqui-López & Cadavid, 2016) data set, 82.94% with M0Droid (Damshenas et al., 2015) data set, and 96.69% with Arslan’s (Arslan, 2021) data set with the proposed method in the study (Şahın, Akleylek & Kiliç, 2022).

Deep learning algorithms, a sub-branch of machine learning, have achieved very good performance results in malware detection. In Arslan’s study called AndroAnalyzer for malware detection, permission-based features were obtained from the original dataset consisting of 7,662 applications and classified with deep neural networks (DNN) (Arslan, 2021). Using this method, 98.16% accuracy was achieved. Xiao et al. trained two different Long-Short Term Memory (LSTM) networks on system call indexes and performed a similarity-based classification process. They obtained 93.7% accuracy with this method (Xiao et al., 2019).

CNN is the most popular deep learning algorithm. The first part of the CNN architecture consists of the feature extractor convolution and pooling layers, and the second part consists of the deep neural network. CNNs have achieved tremendous results in image analysis in recent years (Adegun & Viriri, 2020). Many studies have been carried out on android malware detection by taking advantage of the power of CNNs.

Yadav et al. trained and tested 5,986 images with the EfficientNet-B4 CNN architecture, containing the image representations of the dex extension files of android applications. As a result of the test, 95.7% accuracy was obtained (Yadav et al., 2022). Yen and Sun analyzed the APK file of 1,440 good and bad applications and converted these features into images using the word weighting method TF-IDF. They classified the images they obtained using CNN and reached 92% accuracy (Yen & Sun, 2019). Table S1 summarizes the machine learning-based studies for Android malware detection.

### Fuzzy logic based studies

For fuzzy logic-based malware detection, researchers generally use the ANFIS model. For the training of the ANFIS model, methods based on reducing permission-based features are presented.

Arif et al. proposed a mobile malware detection system based on risk assessment using fuzzy analytical hierarchy process (AHP). They extracted permission-based features from 10,000 malicious applications they obtained from the Drebin and AndroZoo datasets. Out of 274 extracted features, 20 features were selected using the Information Gain method. In the last stage, they divided them into four different risk levels with Fuzzy AHP and reached an accuracy of 90.54% (Arif et al., 2021).

Altaher proposed an evolving hybrid neurofuzzy classifier (EHNFC) for cloaked malware detection. This classifier can change its structure by learning fuzzy rules according to new malware it sees. In order to train and test the proposed model in the study, 250 samples from the GNOME project dataset and 50 permission-based features were extracted from 250 samples downloaded from the Google Play Store. The feature selection process was carried out by applying the information gain method to the extracted features. The proposed approach produced 90% accuracy as a result of the study (Altaher, 2017).

Afifi et al. presented a hybrid approach combining ANFIS model and Particle Swarm Optimization (PSO) for the detection of mobile malware in their study using the dynamic analysis method. Network traffic movements were captured by running 1000 malicious and 20 good applications for 30 min. They obtained 0.4113 RMSE and 0.7721 R2 values with the proposed method in the study carried out on the network traffic movements in which the feature selection process was performed (Afifi et al., 2016).

Altaher & Barukab (2017) proposed an adaptive neuro-fuzzy inference system (FCM-ANFIS) model with fuzzy c-means clustering for Android malware classification. The researchers, who focused on permission-based features in their study and obtained 24 features with the information gain method, reached 91% accuracy.

Abdulla & Altaher (2015) used permission-based features of 200 applications for mobile malware detection. They selected 24 features by applying the information gain method to the datasets with 50 permission-based features. To train these 24 features with the ANFIS model, they divided them into three groups and converted them into one byte format. They used KNN-based fuzzy clustering method together with ANFIS in their model. In the final stage, they trained the proposed model with a data set with three features and one output. As a result of the test, they obtained 75% accuracy. Table S2 presents a summary of fuzzy logic-based studies for Android malware detection.

## Material & Method

In this section, the data set used in the study, the pre-processes performed, the details of the proposed model and the metrics used to evaluate the model are explained.

### Dataset

Two different datasets were used for the study. The malicious application examples in the first dataset were taken from the open-source Drebin (Arp et al., 2014) dataset. The Drebin dataset contains a total of 5,560 malware from 179 malware families. The malicious application examples in the second dataset were taken from the open source CICMalDroid 2020 (Mahdavifar et al., 2020) dataset. The CICMalDroid dataset contains 17,341 applications in five different categories. Examples of benign applications were downloaded from the APKPure web page, which also includes popular applications on the Google Play Store.

All downloaded applications were tested with the VirusTotal (VT Team, 2020) application, as benign samples do not go through the detailed security process while uploading to the Google Play Store. As a result of the VirusTotal scan, 158 applications that were considered malicious by at least one antivirus program for all applications were removed from the dataset. There were problems in accessing the source code or manifest.xml file of some applications, both malicious and benign. These applications were determined and removed from the data set before the feature extraction phase. From the remaining applications in both classes, 250 malicious and 250 benign applications were taken to be used in the study.

The number of samples and the source of the applications used in the study are given in Table 1.

### Proposed method

**Figure 1:**
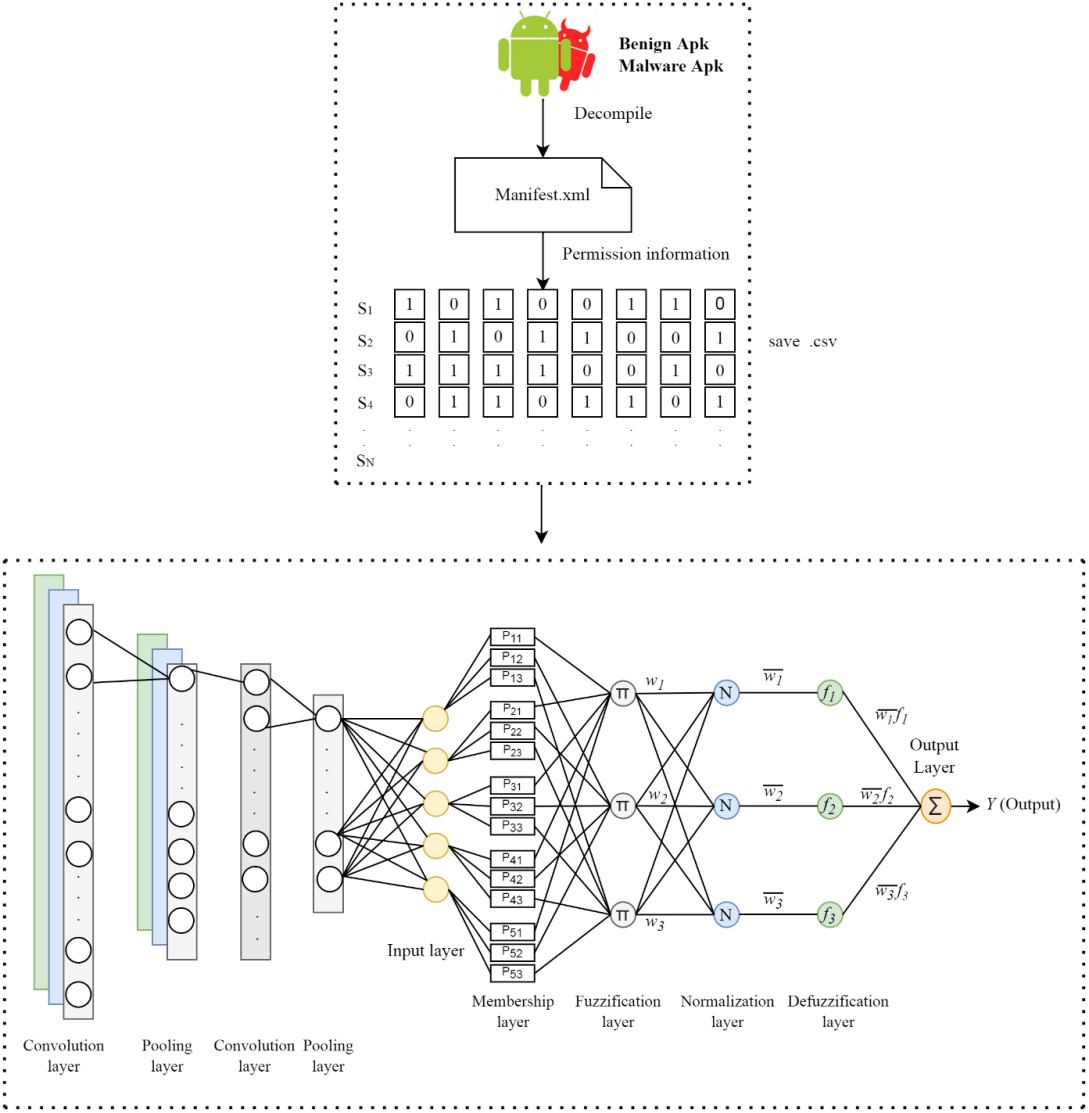
Architecture of the proposed method.

In this study, a hybrid method that combines the convolution layers in the CNN architecture and the ANFIS model is proposed to detect Android malware. In the proposed method, the apk files of the applications are first resolved by reverse engineering. After this process, the manifest.xml file in the apk file is accessed. Permission information of applications is obtained from this file and these information vectors are labeled according to the class of the application. The process of extracting feature is performed using convolution and pooling on the permission information of the applications. The extracted features are sent to the fully connected layer consisting of five neurons. At the last stage, five features obtained from neurons for each application are given as input to the ANFIS model and the prediction is performed. The structure of the proposed model is shown in Fig. 1.

**Table 1:** Number of samples and sources used in the study.

	Class	Number of samples	Source
First dataset	Malware	250	Drebin
	Benign	250	Google Play Store
Second dataset	Malware	250	CICMalDroid
	Benign	250	Google Play Store

### APK decompile

APK, short for Android Package Kit, is a file format used to distribute and install Android applications. An APK file can be considered a package that contains all the necessary items to install on your device. In APK files, there are files and folders containing many data such as source codes of the application, libraries, permission information. The application’s cookies and permissions are in the manifest.xml file, and the compiled java classes are in the classes.dex file. Resources.arsc holds compiled resources used by the application, such as strings. META-INF contains the signature file and a list of sources in the archive. The lib file contains native books that run on a particular architecture of the device. For feature extraction from the application, the APK file, which is the archive, must be decomposed by reverse engineering. In this way, access to the relevant folders can be achieved. The jadx module was used for this process. Jadx compiles .class and .jar files, but can also generate Java source code from Android Dex and Apk.

### Feature extraction and selection

The manifest.xml file in the apk file, which is resolved for feature extraction, is accessed. This xml file contains the permission information requested by the application. For permission information, a list consisting of 325 permission information is used in the first stage. First of all, these permissions are accessed in the application and if there are other permissions requested by the relevant application, the permission list is updated accordingly. The same process is applied for the next application and continues in this way for all samples. A csv file consisting of the permission information of the applications is created by giving a value of 1 if an application requests the relevant permission and 0 if it does not.

### Convolutional layer

After obtaining the permission information of all applications, feature extraction is performed using convolution layers. The convolution operation is performed using filters called kernels. The values in the cells of the kernel represent the weight matrix. The kernels are hovered over the input information according to the determined step amount. At each step, each weight in the kernel is multiplied by the corresponding input values. The output value is obtained by summing the new product values obtained up to the kernel size and it is written to the output matrix. The convolution process is shown in Fig. S1 (Peltarion, 2022a). For the proposed model, two convolution and two pooling layers are used in the feature extraction stage. While the filter size of the first convolution layer is 7 × 1, the filter size used in the second convolution layer is 5 × 1. The number of steps is entered as one in both layers. The weight values on the kernel were randomly determined in the range of 0-1 using the RandonUniform function in the Keras library. The Rectified Linear Unit (ReLU) was used for the activation of the information coming out of the convolution layers.

### Pooling layer

The pooling layer allows reducing the size of the output of the convolution layer. This layer is used to reduce the amount of parameters and computation in the network. It provides a smaller size representation of a sample in the network without losing its distinctive features.

In this layer, as in the convolution layer, navigation is made with the number of steps determined on the input information matrix by using a kernel. However, the kernel used in this layer does not have numerical values and is empty. The kernel records the input values it is on in the matrix and obtains subsets from these input values. In the last step, the one with the highest value from each subset is selected and printed in the relevant area of the output matrix. Maximum pooling and average pooling methods are commonly used in the pooling layer. Maximum pooling was used in this study. The maximum pooling operations are shown in Fig. S2 (Peltarion, 2022b). The kernel size was determined as 2 × 1 and the number of steps was determined as 2.

The information obtained in the last step of the convolution and pooling layers is converted to one-dimensional vector. One-dimensional vectors are used as inputs to the classification network; flatten layer is used for this process.

The layers and parameter values used in the feature extraction process from the permission information of the proposed model are given in Table 2.

**Table 2:** The feature extractor layers of the proposed model.

Layer	Number of Kernels	Size of Kernel/ number of neuron	Stride	Hyperparameters	Activation
Conv2d_1	3	7 × 1	1	RandomUniform Min:0–Max:1	ReLU
Maxpooling2d_1		2 × 1	1		
Conv2d_2	1	5 × 1	1	RandomUniform Min:0–Max:1	ReLU
Maxpooling2d_2		2 × 1	2		
Flatten		–	–	–	–
Dense		5	–	RandomUniform Min:0–Max:1	ReLU

### ANFIS

In the study, an adaptive network-based fuzzy inference system (ANFIS) model was used to classify the features obtained.

ANFIS is a model based on the Takagi-Sugeno fuzzy inference system that combines fuzzy logic and artificial neural networks developed in 1993 (Jang, 1993). This model, which combines the learning ability of artificial neural networks with the decision-making power of fuzzy logic, uses hybrid learning for the optimization of the network. Hybrid learning is a learning approach that consists of back propagation and least squares methods.

Takagi-Sugeno uses the if-then inference rule. The if part of the rule is called the premise, and the then part is called the conclusion. The Takagi-Sugeno rule is defined as follows:

(1) \begin{eqnarray*}IF~{x}_{1}~is~{A}_{1}~\mathrm{{\XMLAMP}}~{x}_{2}~is~{A}_{2}\ldots .\mathrm{{\XMLAMP}}~{x}_{n}~is~{A}_{n}~THEN~y=f({x}_{1},{x}_{2},\ldots .{x}_{n}).\end{eqnarray*}

*x*1, *x*2 and *xn* given in the formula: indicate the input variables. *A*1, *A*2 and An are fuzzy sets obtained by applying a membership function, which defines how each entry point is mapped to a membership value between 0 and 1. The choice of membership function depends on the problem. If *y* is a constant, Takagi-Sugeno is said to be a zero-order Sugeno type, and if *y* is a first-order polynomial, it is said to be a first-order fuzzy type:

(2) \begin{eqnarray*}y={k}_{0}+{k}_{1}{x}_{1}+{k}_{2}{x}_{2}+\ldots ..+{k}_{n}{x}_{n}.\end{eqnarray*}

**Figure 2:**
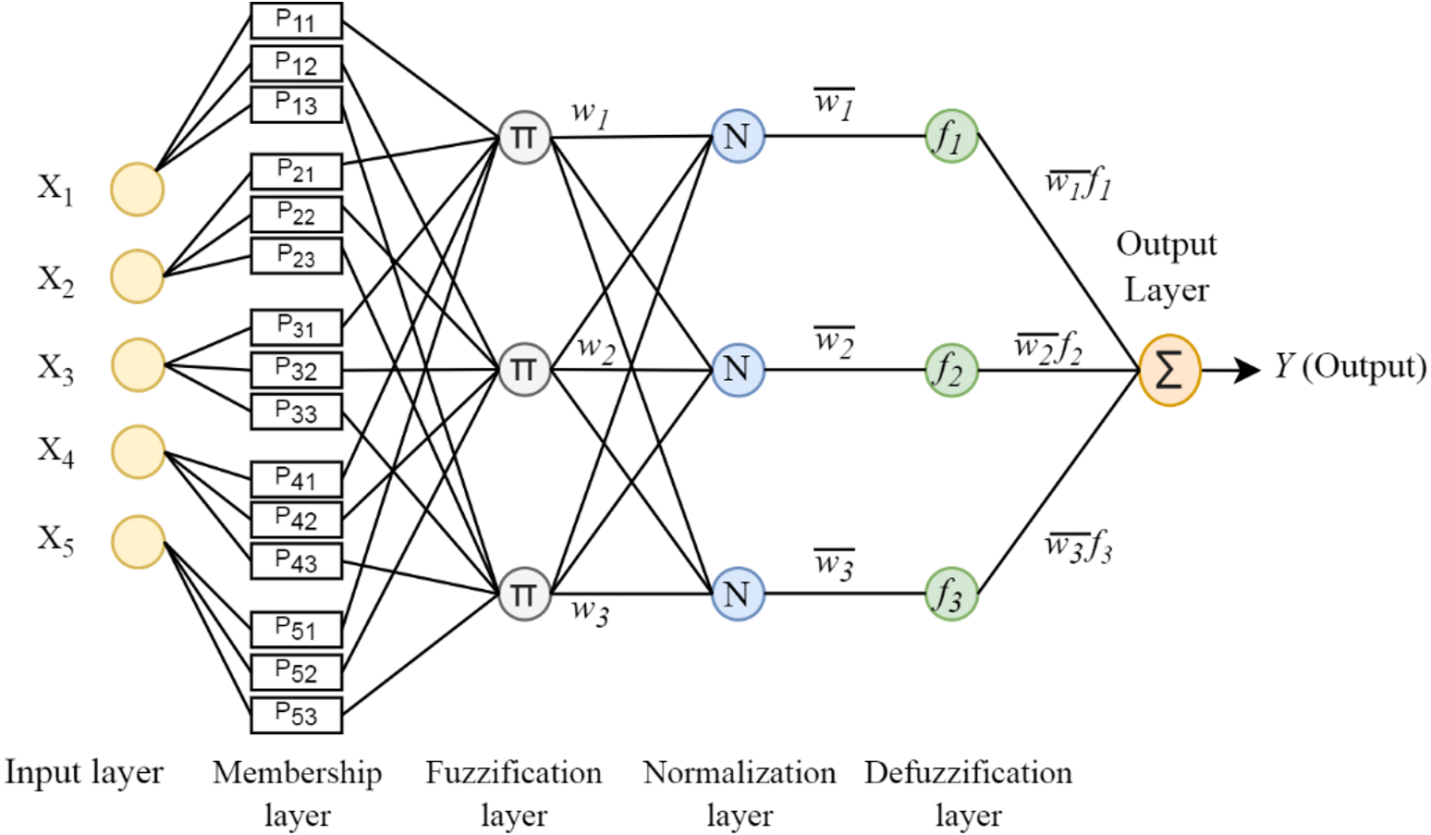
ANFIS architecture.

As shown in Fig. 2, ANFIS architecture consists of 5 layers. Each layer contains a certain number of neurons and performs certain tasks.

Layer 1: It is the fuzzification layer and calculates the fuzzy membership degrees of the inputs.

(3) \begin{eqnarray*}{o}_{i}^{1}={\mu }_{{A}_{i}}(x).\end{eqnarray*}

Layer 2: It is the rule layer. Each neuron in this layer represents a Takagi-Sugeno fuzzy rule. The nodes multiply the information from the previous layer and produce the output value. The output of each node gives the firing strength for each rule.

(4) \begin{eqnarray*}{o}_{i}^{2}={w}_{i}={\mu }_{{A}_{i}}(x)x{\mu }_{{B}_{i}}(x), i=1,2.\end{eqnarray*}

Layer3: It is the normalization layer. The neurons in this layer obtain the normalized firing strength by dividing the firing strength of each rule by the sum of all rules.

(5) \begin{eqnarray*}{o}_{i}^{3}={\overline{w}}_{\mathrm{null}}= \frac{{w}_{i}}{{w}_{1}+{w}_{2}} , i=1,2.\end{eqnarray*}

Layer 4: It is the defuzzification layer. All defuzzification nodes in this layer calculate the output value obtained from the inference of the rules.

(6) \begin{eqnarray*}{o}_{i}^{4}={\overline{w}}_{\mathrm{null}}{f}_{i}={\overline{w}}_{\mathrm{null}}({p}_{i}x+{q}_{i}y+{r}_{i}), i=1,2.\end{eqnarray*}

Layer 5: It is the output layer. It obtains the output value by summing all the signals from the previous layer.

(7) \begin{eqnarray*}{o}_{i}^{5}={\sum \nolimits }_{i}{\overline{w}}_{\mathrm{null}}{f}_{i}= \frac{\sum _{i}{w}_{i}{f}_{i}}{\sum _{i}{w}_{i}} , i=1,2.\end{eqnarray*}

### Evaluation metrics

The confusion matrix is widely used to determine the performance of the models in the classification task. In binary classification tasks, the confusion matrix consisting of a 2 × 2 matrix shows the actual values of the images and the values predicted by the classifier. TP indicates correctly predicted positive results, FP indicates incorrectly predicted positive results, TN indicates correctly predicted negative results, and FN indicates incorrectly predicted negative results. In this study, Accuracy, AUC score, Precision, Recall, and *F*-score metrics were calculated using the parameters obtained from the confusion matrix and presented in the Results section. In addition, Mean Absolute Error(MAE), Mean Square Error(MSE), Root Mean Square Error (RMSE) and R^2^ metrics were calculated and compared with existing studies to measure the distance between the value predicted by the classification model and the true value. The explanations and formulas of the calculated metrics are given in Table S3.

ROC Curve and AUC Score: The ROC curve is a graph showing the performance of the classification model. There is a false positive rate (FPR) on the horizontal axis of the graph and a true positive rate (TPR) on the vertical axis (Adegun & Viriri, 2020). The area under the ROC curve shows the area under curve (AUC) score. The AUC score shows how well the classification model can distinguish between positive and negative samples. As the area increases, the discrimination ability increases.

## Results and Discussion

In this study, two different datasets were used to detect Android malware through a hybrid model based on CNN network and ANFIS. The applications in the first dataset were taken from Drebin, and the applications in the second dataset were taken from the CICMalDroid 2020 dataset. Good apps were obtained from Google Play Store. Permission information was obtained from the manifest.xml file of the applications, and features were extracted using two convolution and two pooling layers in this information. ReLU is used for activation in convolution layers. With the obtained features, the ANFIS model was trained and predictions were made on the test set. In the study 85% of the data sets was reserved for training and 15% for testing. ReLU is used for activation in convolution layers. The predictions obtained from the ANFIS model for the classification problem are set to class 0 if *y* < 0.5, and class 1 if *y* >  = 0.5.

### First dataset results

**Figure 3:**
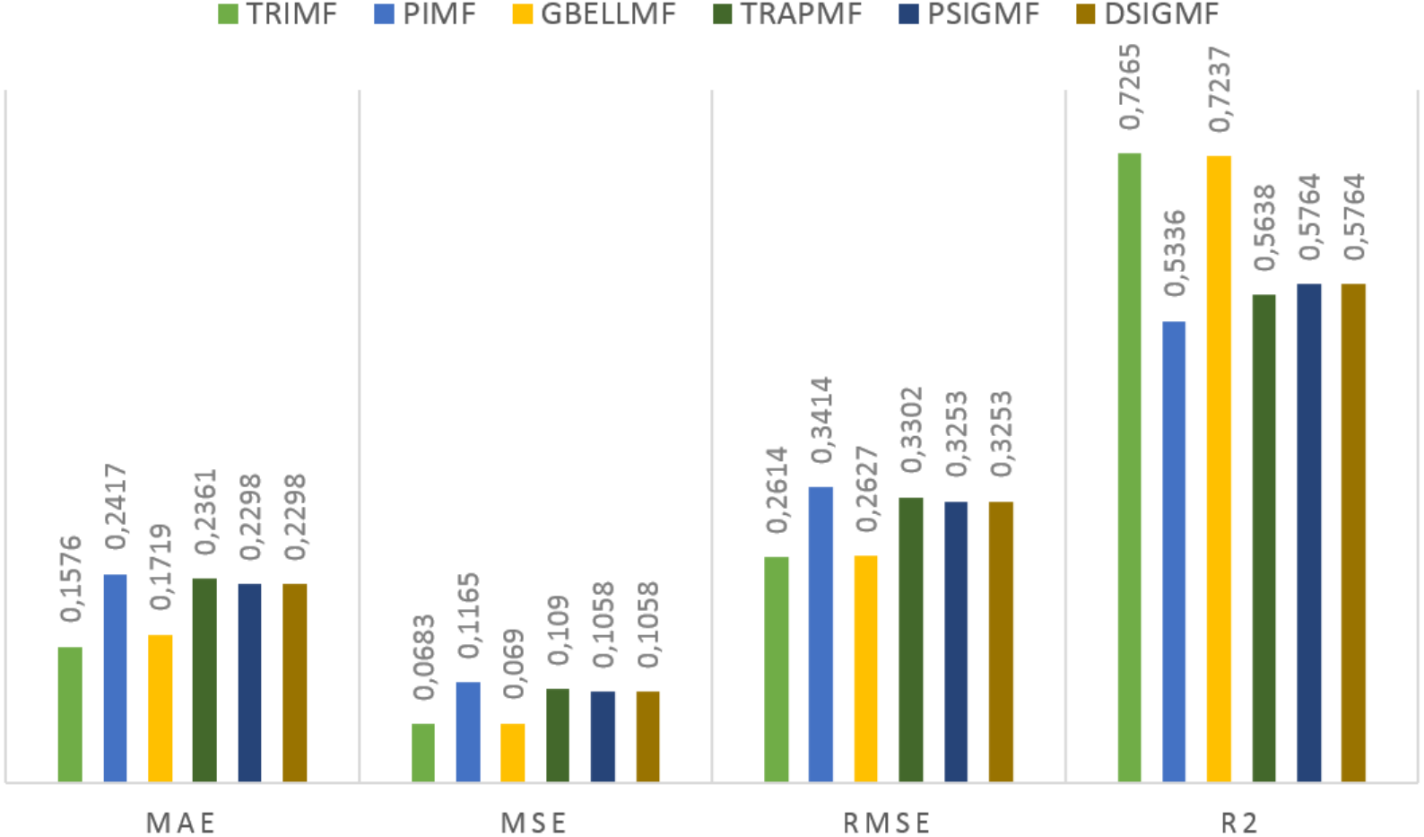
The results of the proposed model for different membership functions.

Under this title, the results obtained on the first data set are presented. The estimation values obtained in the study are shown in Fig. 3 for each membership function used. Using the proposed method, 0.7265 R2, 0.2614 RMSE, 0.0683 MSE and 0.1576 MAE values were reached in the ANFIS model. The most successful prediction values were obtained with the TRIMF membership function. The most unsuccessful results belong to the PIMF membership function. The GBELL function showed close estimation values to the TRIMF function. The estimation values of TRAPMF, PSIGMF and DSIGMF membership functions are very close to each other.

The classification results obtained with the method proposed in the study and the results obtained with the classical machine learning algorithms are given in Table 3. The ANFIS model was trained and tested with the top five features selected from the permission features. TRIMF was used for the membership function.

**Table 3:** Classification results. Bold text shows the best results.

Model	Accuracy	Precision	Recall	*F*-Score	AUC
LDA	0.89	0.8933	0.8933	0.8933	0.8928
SVM	0.86	0.8776	0.8667	0.8665	0.8714
Gaussian Naive Bayes	0.59	0.6903	0.5867	0.4955	0.5589
ExtraTreesClassifier	0.89	0.8933	0.8933	0.8933	0,8928
Decision Tree	0.89	0.8941	0.8933	0.8931	0.8910
KNN	0.85	0.8677	0.8533	0.8530	0.8589
Xgboost	0.92	0.9200	0.9200	0.9200	0.9196
ANFIS	0,90	0,9098	0,9067	0,9068	0.9071
Proposed Method	**0.92**	**0.9215**	**0.9200**	**0.9201**	**0.9196**

**Table 4:** Classification report of the proposed method.

		**Precision**	**Recall**	***F*-Score**
Proposed Method	0	0.8919	0.9429	0.9167
	1	0.9474	0.9000	0.9231
	Macro avg.	0.9196	0.9214	0.9199
	Weighted avg.	0.9215	0.9200	0.9201

In experiments with classical machine learning algorithms, 89% accuracy was achieved with LDA and Decision Tree. The *F*-score of the LDA algorithm was 89.28%, and the *F*-score of the Decision Tree algorithm was 88.10%. While 85% accuracy was obtained with the KNN algorithm, the *F*-score value was 85.3%. With the SVM algorithm, 86% accuracy and 86.65% *F*-score were obtained. The precision value of the SVM algorithm draws attention with 87.7%. The ExtraTreesClassifier, Decision Tree, and XGboost classifiers achieved better results than others. ExtraTreesClassifier reached 89% accuracy, Decision Tree 89% accuracy, and Xgboost algorithm 92% accuracy. Among the classical machine learning algorithms, the Gaussian naive Bayes algorithm gave the most unsuccessful result with 59% accuracy. The ANFIS model, which was tested with the five best-valued permissions features selected, achieved 90% accuracy and 90.68% *F*-score. The proposed model achieved 92% accuracy, 92.15% precision, 92% recall, and 92.01% *F*-score. The classification report of the proposed method is given in Table 4.

Using the proposed model on the first data set, 89.1% precision, 94.2% recall and 91.6% *F*-score were obtained in the benign class. In the malware class, 94.7% precision, 90% recall and 92.31% *F*-score were obtained. The data set used in the study is balanced in terms of benign and malware samples. 91.99% *F*-score was found on the macro average, and 92% *F*-score on the weighted average. The results show that the proposed model can discriminate between benign and malware samples at the same rate.

**Figure 4:**
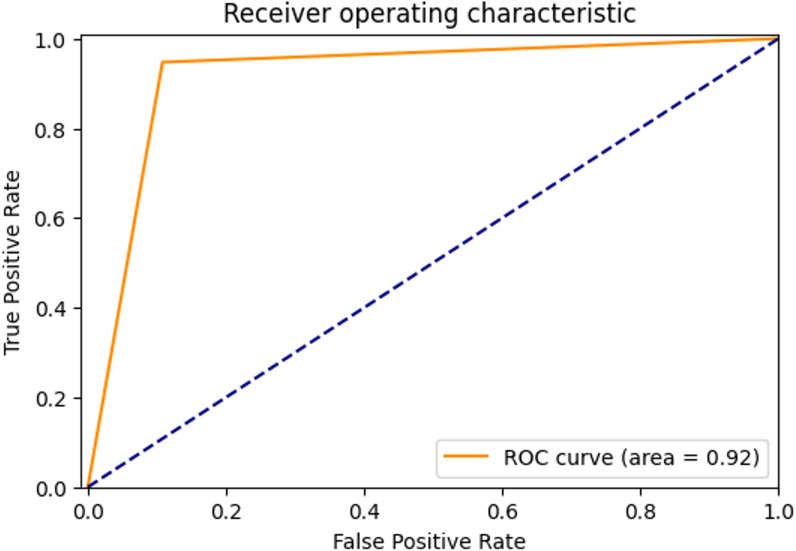
ROC Curve of proposed method.

The ROC curve showing the true positive rate and false positive rate of the proposed method is shown in Fig. 4. The AUC score of the model with strong discrimination ability was found to be 92%.

### Second dataset results

**Figure 5:**
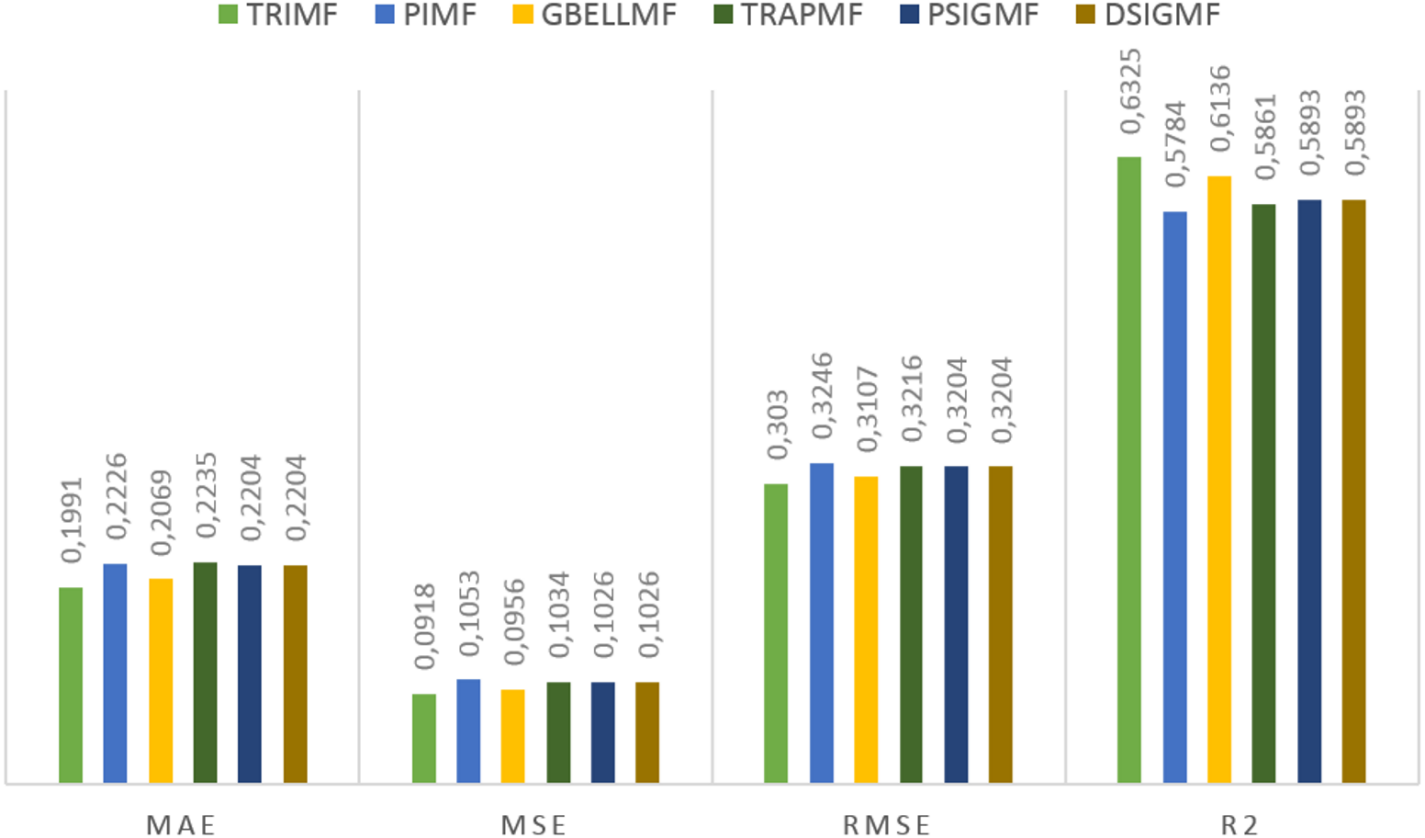
The results of the proposed model for different membership functions.

Under this title, the results obtained on the second dataset are presented. The estimation values obtained in the study are shown in Fig. 5. for each membership function used. Using the proposed method, 0.6325 R2, 0.3030 RMSE, 0.0918 MSE and 0.1991 MAE values were reached in the ANFIS model. The most successful estimation values on the second dataset were obtained with the TRIMF membership function. The most unsuccessful results belong to the PIMF membership function, as in the first dataset. The GBELL function showed close estimation values to the TRIMF function. The estimation values of TRAPMF membership function are lower than PSIGMF and DSIGMF membership functions. PSIGMF and DSIGMF estimated the same values in this dataset.

The classification results obtained with the method proposed in the study and the results obtained with the classical machine learning algorithms are given in Table 5. The ANFIS model was trained and tested with the top five features selected from the permission features. TRIMF was used for the membership function.

**Table 5:** Classification results. Bold text shows the best results.

Model	Accuracy	Precision	Recall	*F*-Score	AUC
LDA	0.7733	0.7739	0.7733	0.7735	0. 7732
SVM	0.8933	0.9132	0.8933	0.8929	0.9
Gaussian Naive Bayes	0.84	0.8681	0.8400	0.8386	0.8482
ExtraTreesClassifier	0.8533	0.8538	0.8533	0.8534	0,8535
Decision Tree	0.8267	0.8279	0.8267	0.8259	0. 8232
KNN	0.8667	0.8857	0.8667	0.8661	0.8732
Xgboost	0.8400	0. 8404	0. 8400	0.8396	0.8375
ANFIS	0,9333	0,9409	0,9333	0,9326	0.9431
Proposed Method	**0.9467**	**0.9478**	**0.9467**	**0.9466**	**0.9487**

The performance of classical machine learning algorithms in the second dataset is low compared to the first dataset. LDA algorithm gave the lowest classification value with 77.3% accuracy. The *F*-score value of this algorithm is 77.3%. The accuracy of the SVM algorithm has increased compared to the first dataset. While the accuracy value and *F*-score value of the SVM algorithm was 89.3%, the AUC score was 90%. Gaussian naive Bayes algorithm obtained the lowest results on the first dataset. In the second dataset, the accuracy value is 84% and the *F*-score value is 83.8%. While the XGboost algorithm showed the best performance among the machine learning algorithms in the first dataset, it showed a lower performance in the second dataset. The accuracy and *F*-score values of the Xgboost algorithm are the same as the Gaussian naive Bayes algorithm. The performance of ExtraTreesClassifier and Decision Tree algorithms decreased in the second dataset. While the accuracy and *F*-score values of the ExtraTreesClassifier algorithm are 85.3%, the accuracy and *F*-score values of the Decision Tree algorithm are 82.6%. The performance of the KNN algorithm increased in the second dataset, and the accuracy value increased to 86%. The performance of the classical ANFIS model and the proposed model increased on the second dataset. The ANFIS model achieved an accuracy of 93% and an AUC score of 94.3%. The proposed model achieved 94.6% accuracy and 94.8% AUC score on the second dataset. The precision value of the proposed model is 94.7%, the recall value is 94.6% and the *F*-score value is 94.6%. The classification report of the proposed method is given in Table 6.

**Table 6:** Classification report of the proposed method.

		**Precision**	**Recall**	***F*-Score**
Proposed Method	0	0.9268	0.9744	0.9500
	1	0.9706	0.9167	0.9429
	Macro avg.	0.9487	0.9455	0.9464
	Weighted avg.	0.9478	0.9467	0.9466

Using the proposed model on the second dataset, 92.6% precision, 97.4% recall and 95% *F*-score were obtained in the benign class. In the malware class, 97% precision, 91% recall and 94.2% *F*-score were obtained. Macro average was 94.6% *F*-score, while weighted average 94.6% *F*-score was found. The results show that the proposed model in the second dataset, as in the first dataset, can distinguish benign and malware samples at the same rate.

**Figure 6:**
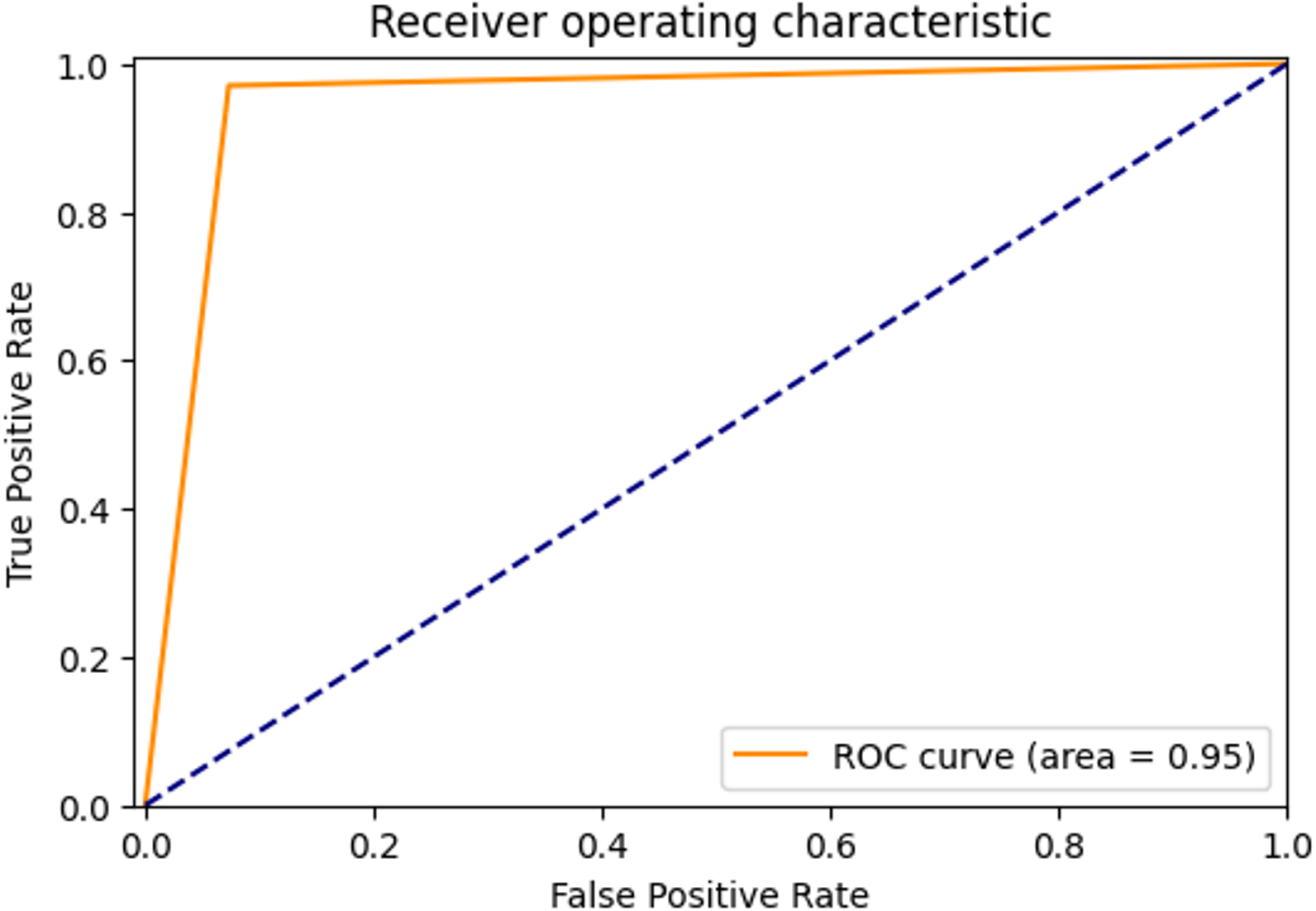
ROC Curve of proposed method.

The ROC curve showing the true positive rate and false positive rate of the proposed method is shown in Fig. 6. The AUC score of the model with strong discrimination ability was found to be 94.87%.

The number of fuzzy logic-based studies for Android malware detection is not enough. ANFIS model is used in most of the studies using fuzzy logic. Since the ANFIS model is rule-based, too many features cause a high number of rules. This causes excessive memory consumption. For this reason, researchers use permission information in ANFIS-based studies and perform the process of choosing the features with the best value. Choosing an attribute from permission information causes hundreds of permission information to be ignored and not taken into consideration. In the model proposed in this study, feature extraction was carried out using convolution and pooling layers from all of the permission information. In this way, the permission information of the applications is not ignored.

Table 7 shows android malware detection studies using fuzzy logic. Detailed information about these studies is given in Section 2.

**Table 7:** Fuzzy logic based studies.

Author	Feature extraction	Feature selection	Classification model	Classification result
Juliza Muhamad Arif	Permission	İnformation Gain	Fuzzy AHP	%90.54 Acc
Altaher	Permission	İnformation Gain	EHNFC	%90 Acc
Afifi et al.	Network traffic	ClassifierSubsetEval	ANFIS + PSO	RMSE 0.4113
Altaher & Barukap	Permission	İnformation Gain	FCM-ANFIS	%91 Acc
Abdulla & Altaher	Permission	İnformation Gain	k-ANFIS	%75 Acc
Proposed Method (First dataset)	Using Convolution layers from permission information	–	ANFIS	%92 Acc
Proposed Method (Second dataset)	Using Convolution layers from permission information	–	ANFIS	%94.66 Acc

Looking at Table 7, the EHNFC model using the ANFIS model achieved 90% accuracy, the k-ANFIS model 75%, and the FCM-ANFIS model 91% accuracy. Afifi et al. used ANFIS and PSO algorithm together to reach 0.4113 RMSE in their study in which they performed dynamic analysis. Arif used Fuzzy AHP for malware detection and achieved 90.54% accuracy. It is seen that these studies, which are carried out using fuzzy logic, have achieved good results in malware detection. The results we obtained with the model we proposed in our study were better than those of other studies. When the values obtained as a result of our study are compared with fuzzy logic-based studies, it has been seen that better results are obtained.

In recent years, good results have been obtained with classical machine learning and deep learning-based studies in Android malware detection. In particular, studies using deep learning provide over 95% accuracy. However, these studies include a high number of parameters and have limitations in terms of memory usage.

Table 8 shows android malware detection studies using deep learning and classical machine learning techniques. Detailed information on these studies is given in Section 2.

**Table 8:** Machine learning based studies.

Author	Classification model	Classification result
Arslan (2021)	DNN	%98.16
Yadav et al. (2022)	CNN(Efficient-B4)	%95.7
Yen & Sun (2019)	CNN	%92
Şahin et al. (2021)	Lineer Regression	%95,6
Xiao et al. (2019)	LSTM	%93.7
Mat et al. (2021)	Naive Bayes	%91,1
Arslan, Doğru & Barişçi (2019)	KNN	%91,95
Proposed Method (First dataset)	ANFIS	%92
Proposed Method (Second dataset)	ANFIS	%94,66

Looking at Table 8, it is seen that deep learning-based studies have achieved good results. Our proposed model, using only two convolution and pooling layers and four cores in total, reached the same value as the work of Yen & Sun (2019), with a low number of parameters. The values we obtained in the second data set are more successful than the work of Yen & Sun (2019). With the CNN (Efficient B4)-based study by Yadav et al. (2022) close values were obtained. The architecture used by Yadav et al. (2022) contains 5,330,571 parameters. In our study, there are 687 parameters in the convolution layers and 243 rules in the ANFIS architecture. The proposed model achieved 92% accuracy in the first dataset and 94.6% accuracy in the second dataset. These results are better than the KNN-based study by Arslan, Doğru & Barişçi (2019). At the same time, it obtained better results than the Bayesian classifier-based study of Mat et al. (2021). Arslan (2021) achieved 98.16% accuracy in his DNN-based study. However, the number of parameters in the model with 4 hidden layers and 300 neurons in each layer is seen as 376,502.

The model proposed in the study has achieved more successful results than fuzzy logic and classical machine learning-based studies, and close results with deep learning-based studies. The advantage of the method we offer is that the permission information of the applications is evaluated without ignoring it. At the same time, our model has low parameter count.

## Conclusion

The Android operating system is open source and free, and its high usage rate compared to other operating systems has made it the target of malicious attackers. Since virus programs are insufficient to ensure the security and privacy of mobile device users using Android, rapid and highly accurate artificial intelligence-based detection systems are needed.

In this study, a hybrid classification method based on CNN’s feature extractor layers and ANFIS is presented for the detection of android malware applications. In the proposed method, apk files are resolved by reverse engineering to reach the manifest.xml file. In this file, the permission information of the applications is obtained and written to the csv file. Convolution and pooling layers in the CNN architecture are used for feature extraction. In the last stage, using the obtained features, predictions are made with the ANFIS model.

The malicious applications used in the study were taken from Drebin for the first dataset and from the CICMalDroid dataset for the second dataset. Good applications were collected from the Google Play Store environment. In order to verify the proposed system, 250 bad and 250 good applications in both datasets and 500 applications in total were used. The classification accuracy and regression results obtained as a result of the experiments were measured with different metrics. With the proposed model, 92% accuracy and 92% *F*-Score value were achieved on the first dataset. On the second dataset, 94.6% accuracy and 94.6% *F*-score were achieved. At the same time, the dataset used in the study was tested with different classical machine learning algorithms and the results were given in a comparison table. It has been seen that the proposed model shows better results in decision making than classical machine learning techniques. Deep learning architectures give good results in malware detection but have limitations due to high parameter count and memory consumption. In the study, comparison tables are given with similar studies based on deep learning. The results obtained showed that similar values were obtained with deep learning methods.

In the present study, malware studies made with the ANFIS model in the literature were examined and compared with the proposed method. In similar ANFIS-based studies, the permission information of the applications was generally used. With the feature selection methods, the columns with the best score of the permission information were selected. In the proposed method, feature extraction is performed with convolution and pooling layers by using all of the permission information of the applications. When the classification results of the proposed model are compared with similar studies, it has been seen that the accuracy and *F*-score value are higher than the others.

In future studies, more efficient results can be obtained by combining the information obtained by static analysis of malicious applications with the information obtained as a result of dynamic analysis.

##  Supplemental Information

10.7717/peerj-cs.1092/supp-1 Supplemental Information 1 The codes for the application’s convolution layers and other parameters

10.7717/peerj-cs.1092/supp-2 Supplemental Information 2 Dataset The dataset contains the permission information of 500 applications. The last column contains the class information. “0” means benign, “1” means malware.
The results of the experiments and comparative analyzes are given in the findings section of the article.

10.7717/peerj-cs.1092/supp-3 Table S1 Summary of machine learning studies for Android malware detection

10.7717/peerj-cs.1092/supp-4 Table S2 Summary of fuzzy logic-based studies for Android malware detection

10.7717/peerj-cs.1092/supp-5 Table S3 Evaluation metrics and definitions used in the study

10.7717/peerj-cs.1092/supp-6 Figure S1 Convolutional layer operations (Peltarion, 2022a; Peltarion, 2022b)

10.7717/peerj-cs.1092/supp-7 Figure S2 Pooling layer operations (Peltarion, 2022a; Peltarion, 2022b)

## Additional Information and Declarations

### Data Availability

The following information was supplied regarding data availability: The code and raw data are available in the Supplemental Files.
