# Supplementary material for: Android malware detection using hybrid ANFIS architecture with low computational cost convolutional layers
Source: PeerJ Comput Sci. 2022 Sep 26;8:e1092. doi: 10.7717/peerj-cs.1092 (PMC9575934; doi:10.7717/peerj-cs.1092)
Supplement: Table S1 [file peerj-cs-08-1092-s003.docx]

Summary of machine learning studies for Android malware detection

| Author | Year | Dataset | Number of samples | Analysis Type | Feature Extraction | Classification Method | Accuracy |
| --- | --- | --- | --- | --- | --- | --- | --- |
| Arslan | 2021 | Drebin, Google Play Store | 7662 | Static | Permission-Based Features | DNN | %98,16 |
| Xiao et al. | 2019 | Drebin, Google Play Store | 7103 | Static | System Calls Features | LSTM | %93,7 |
| Yadav et al. | 2022 | R2-D2 | 5986 | Static | İmage-based features from bytecode | CNN (Efficient-B4) | %95,7 |
| Yen & Sun | 2020 | --- | 1440 | Static | İmage based features from code extracted from APK | CNN | %92 |
| Şahin, Akleylek & Kılıç | 2022 | AMD | 2000 | Static | Permission-Based Features | Lineer Regression | %95,6 |
| Mat et al. | 2021 | AndroZoo  Drebin | 10000 | Static | Permission-Based Features | Naive Bayes | %91,1 |
| Arslan, Doğru & Barışçı | 2019 | Drebin, Google Play Store | 7400 | Static | Permission-Based Features | KNN | %91,95 |
