# Supplementary material for: Android malware detection using hybrid ANFIS architecture with low computational cost convolutional layers
Source: PeerJ Comput Sci. 2022 Sep 26;8:e1092. doi: 10.7717/peerj-cs.1092 (PMC9575934; doi:10.7717/peerj-cs.1092)
Supplement: Table S2 [file peerj-cs-08-1092-s004.docx]

Summary of fuzzy logic-based studies for Android malware detection

| Author | Dataset | Number of samples | Analysis Type | Feature Extraction | Feature Selection | Classification Method | Result |
| --- | --- | --- | --- | --- | --- | --- | --- |
| Arif et al. (2021) | Drebin, AndroZoo | 10.000 | Static | Permission-Based Features | İnformation Gain | Fuzzy AHP | %90,54 |
| Altaher (2017) | GNOME, Google Play Store | 500 | Static | Permission-Based Features | İnformation Gain | Evolving hybrid neurofuzzy classifier (EHNFC) | %90 |
| Afifi et al. (2016) | Google Play, Malgenome | 1220 | Dynamic | Network Traffic Movements | ClassifierSu-bsetEval | ANFIS+PSO | RMSE 0.4113 |
| Altaher & Barukap (2017) | GNOME, Google Play Store | -- | Static | Permission-Based Features | İnformation Gain | Adaptive neuro fuzzy inference system with fuzzy c-means clustering (FCM-ANFIS) | %91 |
| Abdulla & Altaher (2015) | GNOME, Google Play Store | 200 | Static | Permission-Based Features | İnformation Gain | k-ANFIS (k, KNN-based evolving fuzzy clustering (kEFCM) | %75 |
