# Supplementary material for: Android malware detection using hybrid ANFIS architecture with low computational cost convolutional layers
Source: PeerJ Comput Sci. 2022 Sep 26;8:e1092. doi: 10.7717/peerj-cs.1092 (PMC9575934; doi:10.7717/peerj-cs.1092)
Supplement: Table S3 [file peerj-cs-08-1092-s005.docx]

Evaluation Metrics

| Metric | Formula | Description |
| --- | --- | --- |
| Accuracy | $\frac{TP+TN}{TP+FP+TN+FN}$ | It is the ratio of the samples that the classification model predicts correctly to all samples. |
| Sensitivity | $\frac{TP}{TP+FN}$ | Also known as true positive rate. It gives the ratio of correctly predicted positive samples to all actually positive samples. |
| Specificity | $\frac{TN}{TN+FP}$ | It is known as the true negative rate. It gives the ratio of correctly guessed negative samples to all actually negative samples. |
| Precision | $\frac{TP}{TP +FP}$ | It is the ratio of correctly predicted positive samples to all positively predicted samples. |
| F-Score | $\frac{2\times Precision\times Recall}{Precision+Recall}$ | It gives the harmonic average of the recall and precision values. |
| MAE | $\frac{1}{N}$ $\sum_{i=1}^{N} \vert y_{i}-ŷ\vert$ | It gives the mean of the absolute difference between the predicted value and the actual value. . y_i represents the predicted values and ŷ the actual values. |
| MSE | $\frac{1}{N}$ $\sum_{i=1}^{N} {(y_{i}-ŷ)}^{2}$ | It gives the square of the mean difference between the predicted value and the actual value. |
| RMSE | $\sqrt{\frac{1}{N} \sum_{i=1}^{N} {(y_{i}-ŷ)}^{2}}$ | It is used to find the distance between the value predicted by the model for each sample and the actual value. It gives the root value of the mean square error. |
| R^2^ | 1 - $\frac{\sum{(y_{i}-ŷ)}^{2}}{\sum{(y_{i}-ỹ)}^{2}}$ | It shows how well the predicted values fit the actual values. The value obtained between 0 and 1 is interpreted as a percentage. The higher the value, the better the model. |
