# Supplementary figures and images for: Android malware detection using hybrid ANFIS architecture with low computational cost convolutional layers
Source: PeerJ Comput Sci. 2022 Sep 26;8:e1092. doi: 10.7717/peerj-cs.1092 (PMC9575934; doi:10.7717/peerj-cs.1092)

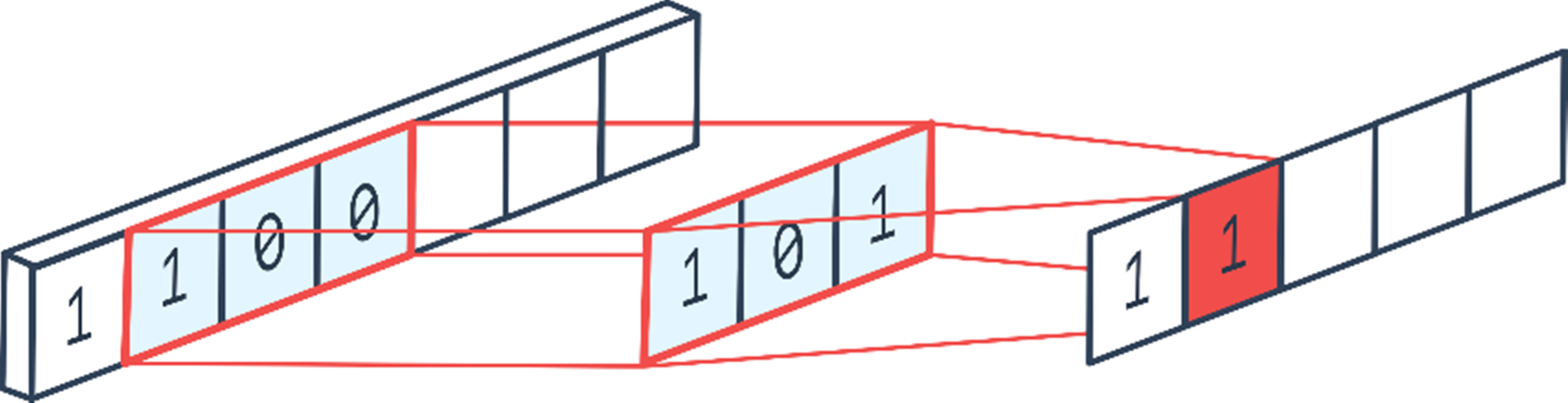

Supplement: Figure S1 [file peerj-cs-08-1092-s006.png]

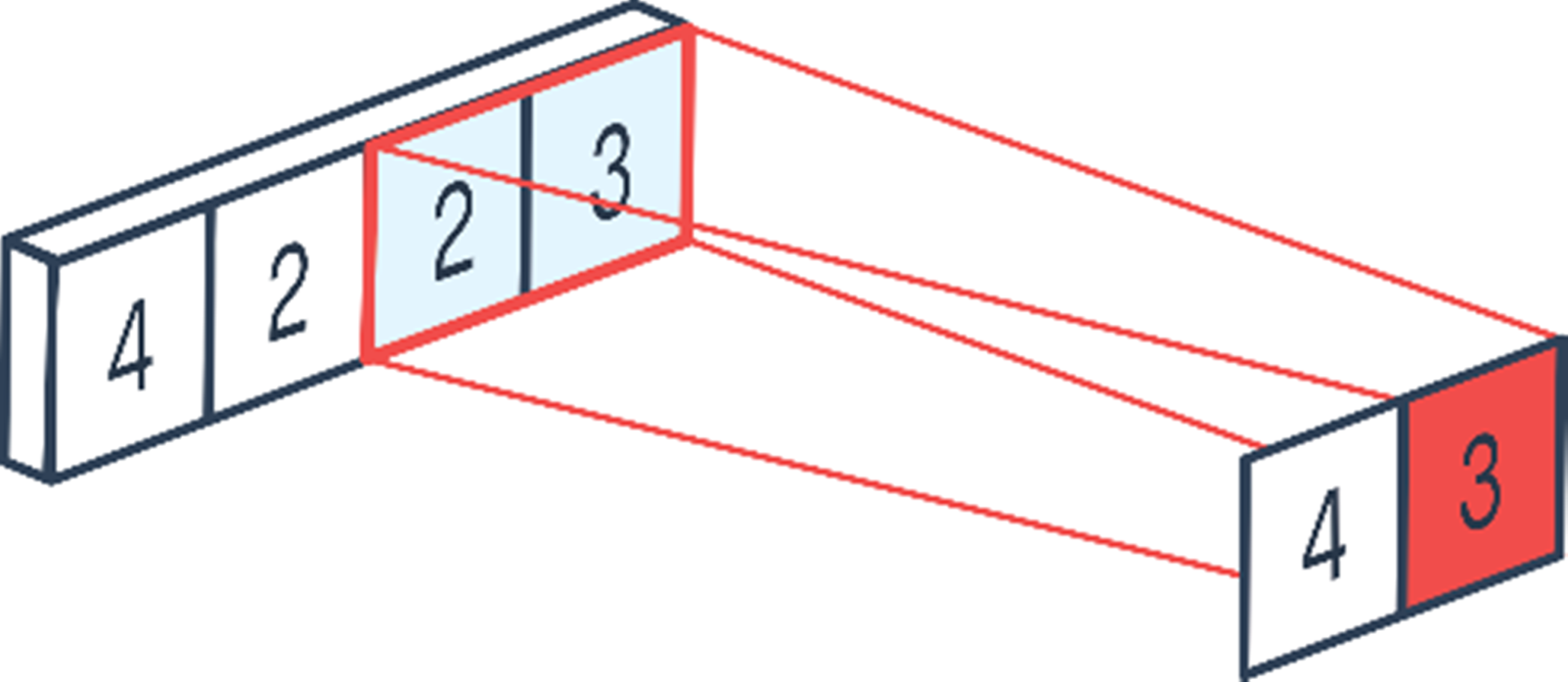

Supplement: Figure S2 [file peerj-cs-08-1092-s007.png]
